# A Retrospective Comparison of Clinical Efficacy between Multimodal Analgesia and Patient-Controlled Epidural Analgesia in Patients Undergoing Total Knee Arthroplasty

**DOI:** 10.3390/medicina59122137

**Published:** 2023-12-08

**Authors:** Teng-Kuan Wang, Yang-Yi Wang, Ming-Chou Ku, Kui-Chou Huang, Kwok-Man Tong, Chih-Cheng Wu, Yuan-Hsin Tsai

**Affiliations:** 1 Department of Orthopedics, Kaohsiung Municipal Gangshan Hospital, Kaohsiung 820002, Taiwan; dog801229@hotmail.com.tw; 2 Department of Orthopedics, Show Chwan Memorial Hospital, Changhua 500009, Taiwan; poker6609@gmail.com (Y.-Y.W.); showjoeku@gmail.com (M.-C.K.); 3 Department of Orthopedics, Asia University Hospital, Taichung 413505, Taiwan; kuichouhuang@gmail.com (K.-C.H.); tongkwokman@gmail.com (K.-M.T.); 4 Department of Anesthesiology, Taichung Veterans General Hospital, Taichung 407204, Taiwan; chihcheng.wu@gmail.com

**Keywords:** total knee arthroplasty, postoperative pain management, multimodal analgesic strategy, patient-controlled epidural analgesia, adverse effects, motor blockade, numbness

## Abstract

*Background and Objectives:* Adequate pain management during early rehabilitation is mandatory for improving the outcomes of patients undergoing total knee arthroplasty (TKA). Conventional pain management, mainly comprising opioids and epidural analgesia, may result in certain adverse effects such as dizziness, nausea, and motor blockade. We proposed a multimodal analgesic (MA) strategy involving the use of peripheral nerve block (NB), periarticular injection (PAI), and intravenous patient-controlled analgesia (IVPCA). This study compared the clinical efficacy and adverse effects of the proposed MA strategy and patient-controlled epidural analgesia (PCEA). *Materials and Methods:* We enrolled 118 patients who underwent TKA under spinal anesthesia. The patients followed either the MA protocol or received PCEA after surgery. The analgesic effect was examined using a numerical rating scale (NRS). The adverse effects experienced by the patients were recorded. *Results:* A lower proportion of patients in the MA group experienced motor blockade (6.45% vs. 22.98%) compared to those in the PCEA group on the first postoperative day. Furthermore, a lower proportion of patients in the MA group experienced numbness (18.52% vs. 43.33%) than those in the PCEA group on the first postoperative day. *Conclusions:* The MA strategy can be recommended for reducing the occurrence of motor blockade and numbness in patients following TKA. Therefore, the MA strategy ensures early rehabilitation while maintaining adequate pain relief.

## 1. Introduction

Total knee arthroplasty (TKA) is a highly effective, successful, and economically efficient intervention for managing advanced knee arthritis. It offers significant improvements in pain alleviation, functional recovery, and overall quality of life [1,2,3,4,5]. The number of patients undergoing TKA is increasing annually in the United States owing to the aging population, which is associated with an increase in end-stage knee osteoarthritis [6,7]. Postoperative pain is a serious problem that patients may experience following TKA, which may result in limited range of motion (ROM), arthrofibrosis, venous thrombosis, pneumonia, poor sleep, and anxiety. These lead to reduced patient satisfaction and quality of life [8,9]. The National Institutes of Health Consensus Statement on Total Knee Replacement recommends mandatory pain management during early rehabilitation to improve the outcomes following TKA [10].

Conventional pain management strategies include the use of oral analgesics, parenteral narcotics, patient-controlled epidural analgesia (PCEA), and intravenous patient-controlled analgesia (IVPCA). Oral analgesics can have various adverse effects, such as gastrointestinal disturbances, nausea and vomiting, drowsiness, constipation, liver or kidney damage, and cardiovascular complications, based on the type and dosage of the medication [11,12]. Many of the aforementioned strategies involve the use of opioids, which may result in the occurrence of certain adverse effects, such as nausea, vomiting, ileus, and pruritis [13,14]. PCEA has been widely used owing to its dominant effect and superior pain relief compared to that of parenteral medication [15]. PCEA occasionally results in motor blockade [16,17], which may further affect rehabilitation. Despite adequate pain control, patients may experience certain discomfort and uneasiness.

Over the past two decades, several innovative pain management strategies, such as nerve block (NB) and periarticular injection (PAI), have emerged for reducing the occurrence of adverse effects. NB is reportedly effective in postoperative analgesia and reduces narcotic use at the expense of other potential problems, such as diminished muscle strength and nerve damage [18,19,20,21,22]. The outcomes following PAI were reportedly encouraging in terms of pain relief, which was associated with lower pain scores, accelerated hospital discharge, and reduced postoperative consumption of analgesics such as opioids [23,24]. However, approximately 50–60% of the patients still required other analgesic modalities for satisfactory pain control [25,26].

Multimodal pain management is a potential solution for minimizing the adverse effects of various pain relief methods. Despite numerous studies on the topic, a consensus on the best multimodal analgesic protocol remains elusive. We proposed a multimodal analgesic (MA) protocol which combined the use of NB, PAI, and IVPAC. It was hypothesized that the present MA protocol leads to fewer adverse effects than traditionally used PCEA and thus enhances postoperative muscle control for ambulation and rehabilitation. The chief aim of this research is to conclusively evaluate these hypotheses and provide an evidence-based recommendation for the perioperative analgesic protocol of TKA.

## 2. Materials and Methods

### 2.1. Study Design and Patient Selection

This retrospective study was approved by the institutional review board of Taichung Veterans General Hospital (IRB TCVGH No: CE17352A). We conducted this retrospective cohort study on the effect of pain management in patients undergoing TKA to determine whether our proposed MA protocol is better than PCEA in terms of analgesia and potential side effects.

**Figure 1:**
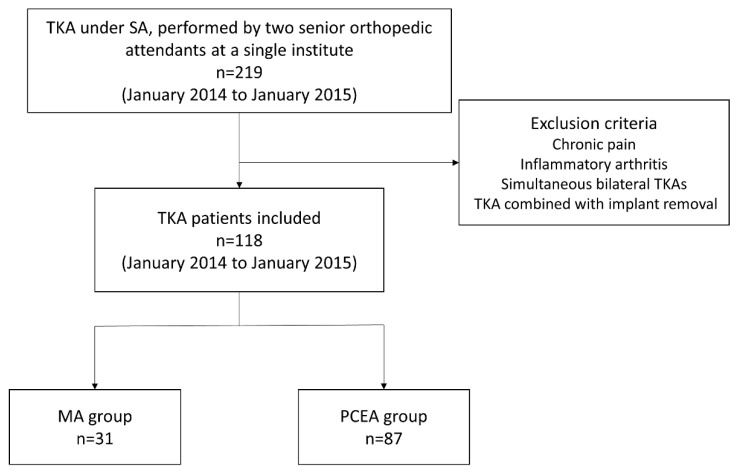
Flowchart for patient selection in this study.

We enrolled 219 patients who had been diagnosed with knee osteoarthritis and were scheduled to undergo TKA. Surgeries were performed by two senior orthopedic attendants at a single institute between January 2014 and January 2015 (Figure 1). All the patients underwent implantation with a Zimmer NexGen Legacy^®^ LPS-Flex Knee (Zimmer Biomet Inc., Warsaw, IN, USA), a popular prosthetic knee design asserting the capability to facilitate high flexion with a minimized risk of edge loading [27]. All the surgical procedures were performed according to the standard guidelines outlined in the procedure book.

The inclusion criteria were as follows:

(1) Patients aged 50–80 years.
(2) Patients diagnosed with advanced knee osteoarthritis with surgical indication.
(3) Patients receiving spinal anesthesia during operation.
(4) Conscious patients with no cognitive impairment.

The exclusion criteria were as follows:

(1) Chronic pain or inflammatory arthritis.
(2) Simultaneous bilateral TKA.
(3) Unilateral TKA combined with implant removal.
(4) Patients receiving PCEA through postoperative supplementation of NB for better pain control.
(5) Patients with missing data necessary for the analysis.

Patients who met all the inclusion criteria were randomly assigned to two different analgesic methods, chosen based on the preference and expertise of the anesthesiologist on duty for that day. We selected 118 patients based on the inclusion criteria; 31 received our proposed MA protocol (MA group) and 87 received only PCEA (PCEA group, comparison group; Table 1 and Figure 1). Two senior orthopedic attendants performed TKA using similar surgical methods, including skin incision, medial parapatellar approach, and surgical techniques for the bone and soft tissue. All the patients underwent implantation with a Zimmer NexGen Legacy^®^ LPS-Flex Knee. All the surgical procedures were performed according to the standard guidelines outlined in the procedure book. All patients underwent standard rehabilitation programs at our institution postoperatively, including ankle pumping, quadriceps setting, and achieving a ROM of up to 90 degrees before being discharged.

### 2.2. Pain Management Strategies

The MA protocol included the administration of ultrasound-guided NB in the femoral and obturator nerves prior to spinal anesthesia; PAI with mixed non-steroidal anti-inflammatory drugs (NSIADs), morphine, ropivacaine, and epinephrine intraoperatively (Table 1); and IVPCA postoperatively. In the ultrasound-guided NB, we first located the femoral nerve, which involved positioning the transducer at the level of the femoral crease, lateral to the pulse of the femoral artery. In adult patients, 10–15 mL of local anesthetic is considered adequate for a successful nerve block. We subsequently identified the obturator nerve branches located at the level of the femoral crease and medial to the femoral vein. Each obturator branch was treated with an injection of 5–10 mL of the local anesthetic solution. NB typically ensures pain relief for approximately 6–12 h. The injection sites for the PAI included the posterior capsule, collateral ligaments, quadriceps tendon, periosteum, and arthrotomy edges. The PAI technique was first described by Guild III [28]. Patients in the PCEA group underwent epidural catheterization prior to spinal anesthesia, and analgesics were administered through a catheter postoperatively using a patient-controlled machine. The details of the PCEA procedure are listed in Table 1. We employed a 4 h limit to restrict the infusion volume, rather than using maximal boluses. Moreover, we employed a 4 h limit instead of a 1 h limit at our hospital.

**Table 1:** MA and PCEA protocols.

	MA	PCEA
Pre-operation	NB Femoral nerve (0.17% bupivacaine + 0.67% xylocaine, 30 mL) Obturator nerve (0.25% bupivacaine, 10 mL)	
Intra-operation	PAI [29] Ropivacaine 100 mg Ketorolac 30 mg Morphine 5 mg Epinephrine 0.1 mg	
Post-operation	IVPCA (fentanyl 4 μg/mL)	PCEA 0.16% ropivacaine with fentanyl 1.5 μg/mL in normal saline PCEA drug: 500 mL/bag Bolus doses: 4–5 mL Background infusion: 4–5 mL/hr Lock out interval: 15–20 mL 4 h limit: 40–55 mL

All the patients underwent TKA under spinal anesthesia at the L3–L4 or L4–L5 interspinous space with 12.5–15 mg bupivacaine (2.5–3 mL 0.5% bupivacaine). MA: multimodal analgesia; PCEA: patient-controlled epidural analgesia; NB: nerve block; PAI: periarticular injection; IVPCA: intravenous patient-controlled analgesia.

All the patients underwent five follow-ups after surgery (the postoperative days (PODs) are mentioned in Table 2).

### 2.3. Data Acquisition and Efficacy Assessments

Data collected from patients’ medical records included sex, age, weight, height, body mass index (BMI), surgery date, and Kellgren–Lawrence grade. Analgesic efficacy was evaluated by measuring the pain intensity using a numerical rating scale (NRS). The NRS scores ranged from 0 (no pain) to 10 (worst pain). We chose the NRS over other pain scales, such as the visual analog scale or verbal rating scale, because it provides a more precise measurement of the pain intensity and is more sensitive to the changes over time. The NRS also facilitates convenient data analysis. The occurrence of adverse effects, including motor blockade, numbness, postoperative nausea and vomiting (PONV), and dizziness, was considered the secondary outcome measure. Three grades of motor blockade were recorded: no block, weakness, and motionlessness.

### 2.4. Statistical Analysis

Statistical analyses were performed using IBM SPSS version 22.0 (IBM, New York, NY, USA). Assuming a normal distribution of patients and random sampling, the baseline demographics and outcomes were compared using independent *t*-tests for continuous variables (age, weight, height, BMI, and pain intensity) and chi-square tests for categorical variables (sex, Kellgren–Lawrence grade, occurrence of motor blockade, numbness, PONV, and dizziness). We applied Yate’s correction or Fisher’s exact test when very few patients were included in a specific category. Yate’s correction aims to rectify the error that arises from the assumption that the discrete probabilities of the frequencies in the table can be approximated by a continuous distribution, such as the chi-squared distribution. A significance level of *p* < 0.05 was considered statistically significant for all tests.

## 3. Results

From the total sample, 31 patients received MA while 87 underwent PCEA. Gender distribution was similar between the two groups, with 80.65% of the MA group being women, compared to 75.86% in the PCEA group; this difference was not statistically significant (*p* = 0.804). The distribution of ages was comparable between the two groups, with the MA group having an average age of 69.35 ± 8.24 years and the PCEA group averaging 70.34 ± 7.05 years. This disparity was not statistically significant (*p* = 0.523). No significant differences were observed in the height and weight of the patients between the MA and PCEA groups. The mean BMI in the MA group was 26.90 ± 5.08, while it was 28.47 ± 5.27 in the PCEA group. This difference was also not statistically significant (*p* = 0.113). Regarding the grading of knee osteoarthritis, in the MA group, 6.45% of patients were classified with Kellgren–Lawrence grade III, while 93.55% were categorized as grade IV. Similarly, in the PCEA group, 10.34% of patients had Kellgren–Lawrence grade III osteoarthritis, while 89.66% were categorized as grade IV. There was no significant difference in the severity of osteoarthritis between the two groups (*p* = 0.759) (Table 3). No significant differences were observed in the NRS scores between the MA and PCEA groups during the first three visits, both at rest and during movement. The MA group exhibited pain control comparable to that of the PCEA group within 24 h postoperatively (Table 4).

**Figure 2:**
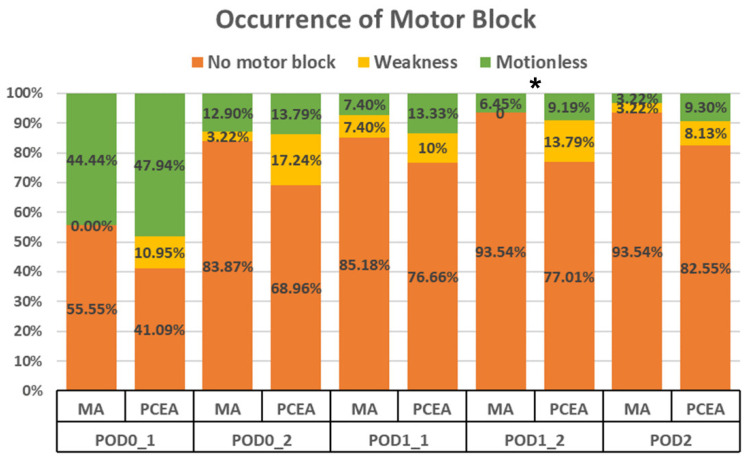
Occurrence of motor blockade in the lower extremities between the multimodal analgesia (MA) and patient-controlled epidural analgesia (PCEA) groups. * *p* < 0.05.

Compared to the PCEA group (20 out of 87), a significantly lower proportion of patients in the MA group (2 out of 31) experienced motor blockade (6.45% vs. 22.98%, *p* = 0.028; Figure 2) during the second visit on POD 1. Furthermore, on POD 2, the MA group demonstrated a trend towards a lower incidence of motor blockade; however, no significant difference was observed (Figure 2).

**Figure 3:**
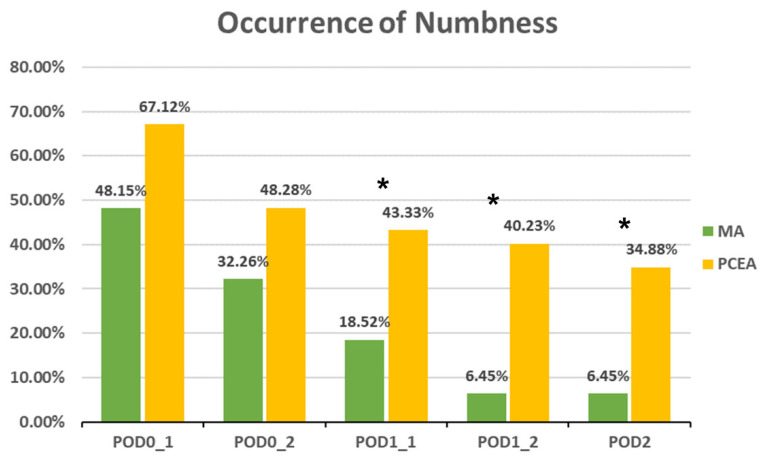
Occurrence of numbness between the multimodal analgesia (MA) and patient-controlled epidural analgesia (PCEA) groups. * *p* < 0.05. Chi-square test.

**Figure 4:**
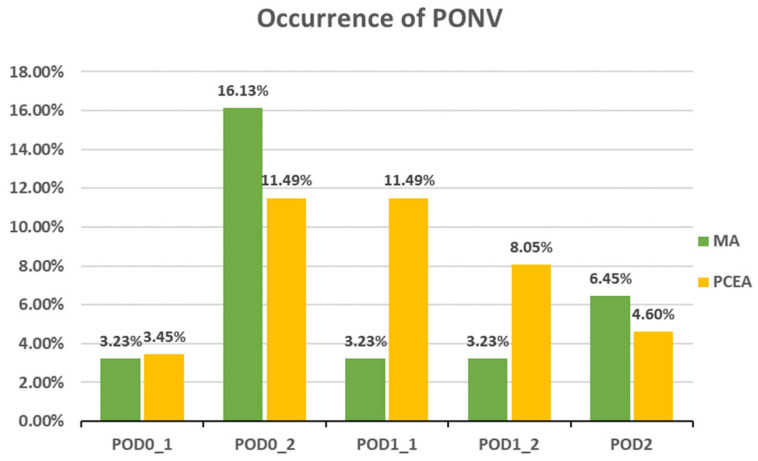
Occurrence of postoperative nausea and vomiting (PONV) between the multimodal analgesia (MA) and patient-controlled epidural analgesia (PCEA) groups. Chi-square test.

**Figure 5:**
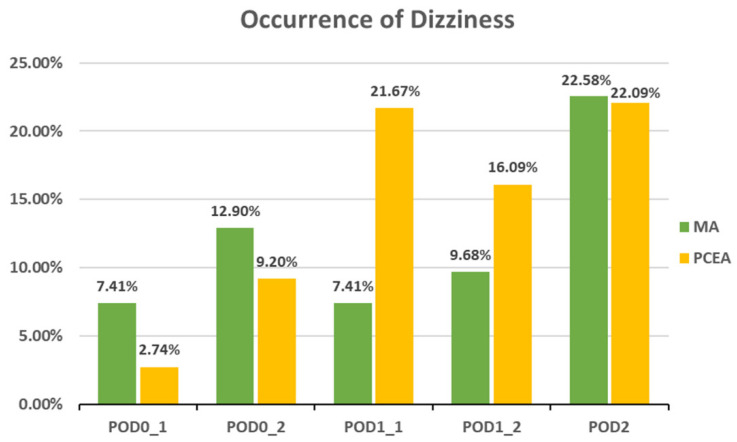
Occurrence of dizziness between the multimodal analgesia (MA) and patient-controlled epidural analgesia (PCEA) groups. Chi-square test.

Compared to the PCEA group, a significantly lower proportion of patients in the MA group experienced numbness after POD 1 (POD1_1: 18.52% (5 out of 27) vs. 43.33% (26 out of 60), *p* = 0.031; POD1_2: 6.45% (2 out of 31) vs. 40.23% (35 out of 87), *p* = 0.000; POD 2: 6.45% (2 out of 31) vs. 34.88% (30 out of 86), *p* = 0.002; Figure 3). In addition, the incidences of PONV and dizziness were lower in the MA group than in the PCEA group on POD 1 during clinical evaluation; however, the differences were not statistically significant (Figure 4 and Figure 5).

## 4. Discussion

The novel MA protocol used in this study resulted in fewer adverse effects from 24 h after the TKA surgery, while maintaining comparable postoperative analgesia with the PCEA group within the first 24 h. This important finding suggests that the MA protocol can not only preserve effective pain relief during the most critical initial 24 h but also contributes to enhanced rehabilitation starting from 24 h postoperatively. IVPCA provided continued pain relief after waning of the analgesic effects on NB or PAI within the initial 24 h. Compared to MA, PCEA was more effective in maintaining continuous analgesia from 24 to 72 h, thereby providing a longer analgesic duration. PCEA may be considered more suitable for patients undergoing intricate joint surgeries, which necessitate comprehensive pain control within the initial 72 h postoperatively, rather than prioritizing early ambulation. This is especially applicable to individuals undergoing revision TKA, simultaneous bilateral TKA, or those with heightened pain sensitivity. However, the incidences of both motor blockade and numbness were higher in the PCEA group than in the MA group, particularly since POD 1. This is probably because the motor blockade and numbness in the MA group were primarily attributed to the NB, and these effects subsided 24 h after surgery. NB can cause motor blockade and numbness because it affects the transmission of signals along the nerve fibers, including motor and sensory neurons. Furthermore, the incidence of both PONV and dizziness was higher in the PCEA group, particularly on POD 1. PCEA can cause PONV and dizziness in some cases owing to sympathetic blockade, opioid medications, or dural puncture.

### 4.1. Comparison with Previous Studies

Postoperative pain following TKA is concerning not only for patients but also for orthopedic surgeons. Adequate pain management not only enables early rehabilitation, but also reduces complications and correlates with patient satisfaction [30]. Mahoney et al. compared the effects of parenteral opioids with those of epidural opioids after TKA and reported that pain relief was achieved in 88% of the epidural analgesia cases compared to 61% of the conventional analgesia cases [16]. PCEA, which is a powerful and effective analgesic method, has been accepted as the gold standard for postoperative analgesia as established by Bromage et al. [31,32]. Nevertheless, opioid-related adverse effects, including nausea, vomiting, dizziness, urinary retention, and respiratory depression remain major concerns [13,33,34].

In recent years, efforts have been made to develop PAI analgesic protocols to improve early postoperative analgesia while reducing the occurrence of associated adverse effects [23,35,36,37]. Tsukada et al. reported that the PAI group exhibited a significantly reduced area under the curve for pain scores at rest compared with the epidural analgesia group (788.0 vs. 1065.9; *p* = 0.0059) [38]. However, they did not report the pain scale considering the patients in motion. Milani et al. conducted a randomized controlled trial and reported that a PAI anesthetic protocol with ropivacaine (1%, 20 mL) did not produce superior results concerning pain, edema, and ROM compared to oral or intravenous analgesia in patients undergoing TKA [39]. Kelley et al. compared different combinations of PAI injections and found that a combination of ropivacaine, epinephrine, clonidine, and ketorolac resulted in stronger early postoperative pain control [40]. Our study modified the PAI regimen reported by Spangehl et al. [29] based on the physical characteristics of Asian patients. Asians typically have smaller body sizes; thus, we reduced the dosage.

The femoral, popliteal, and obturator nerves are the major innervations of the knee joint. Paul et al. [21] reported the efficacy of NB in postoperative analgesia. Femoral NB (FNB) is considered the gold standard for TKA; however, approximately 60–90% of patients still experience severe postoperative pain even after successful FNB [25,41]. Posterior knee pain is predominantly caused by the sciatic and obturator nerves. Seo et al. demonstrated the effectiveness of popliteal sciatic NB in reducing severe early pain following TKA [42]. Subsequently, several studies have compared the analgesic efficacy of NB with that of PAI [43,44,45]. However, these studies reported heterogeneous results. A meta-analysis by Wang et al. indicated that single-shot FNB may offer better pain relief in the early postoperative period than single-shot PAI and that continuous PAI may provide postoperative analgesia comparable to that of continuous FNB [46]. However, no firm conclusions could be drawn owing to the variations in these studies.

### 4.2. Implications of Our Findings

No study has demonstrated the superiority of any analgesic intervention following TKA. MA combined with PAI has received increasing interest; however, no further combinations of preoperative NB or postoperative IVPCA have been reported. The proposed MA strategy implements preoperative NB, intraoperative PAI, and postoperative IVPCA. The protocol is administered through three distinct routes: regional anesthesia, local infiltration anesthesia, and intravenous analgesics, and encompasses various regimens with different pharmacologic mechanisms, including short-duration anesthetics, long-duration anesthetics, NSAIDs, and opioids. The objective of this MA strategy is to decrease the dosage of each analgesic technique while enhancing pain relief through their synergistic or additive effects. This approach may further mitigate the severity of the individual adverse effects associated with each drug. In terms of clinical significance, within the first 24 h postoperatively, MA and PCEA exhibit similar analgesic effects. Consequently, MA, with its lower risk profile, can be considered as a substitute for PCEA. Unlike PCEA, MA does not require invasive epidural procedures and indwelling catheters, reducing the risks of spinal injury and intrathecal infection. With pain scores below one within the initial 24 h, rehabilitation activities, such as ankle pumping and ROM training, can be initiated. Starting from 24 h postoperatively, MA exhibits significantly fewer adverse reactions than PCEA, reducing the need for additional medications to manage adverse effects. Additionally, both motor blockade and numbness are substantially reduced with MA during this period, making it more suitable to initiate activities such as walker ambulation. Patients are more confident in their future walking function, leading to higher satisfaction with the surgery. Despite MA showing significantly higher pain scores compared to PCEA, the pain intensity remains manageable, allowing for the implementation of rehabilitation practices.

### 4.3. Limitations

This study has certain limitations. First, owing to the retrospective nature of the study, patients with missing medical records and unrecorded NRS scores were not included in the study. Additionally, we could not confirm the complete documentation of the adverse effects of all the patients, which could have resulted in potential data analysis inaccuracies. Second, patients might have been prescribed different oral pain medications during their hospitalization, and the variability in the efficacy and potential side effects of these drugs could have contributed to potential information bias. Third, we did not compare the differences in pain relief between NB with IVPCA and PAI with IVPCA. Therefore, whether NB or PAI primarily contributed to the analgesic effect remains uncertain. Lastly, the research was carried out at one hospital in Taiwan. It will be crucial to pursue additional investigations that encompass a broader range of healthcare institutions across various countries and ethnicities.

Based on our inclusion and exclusion criteria, we only recruited patients aged 50–80 years who received spinal anesthesia during surgery. Patients with inflammatory arthritis or those undergoing simultaneous bilateral TKA or unilateral TKA combined with implant removal were excluded. The chosen age limit of 50 to 80 years is due to this age range representing the most common demographic for individuals undergoing TKA surgery, and we aim to understand the response of this population to pain management. Secondly, patients who are too young tend to have greater postoperative activity demands and lower pain tolerance. Also, patients who are too old typically have more comorbidities, which can significantly impact postoperative issues such as weakness, PONV, and dizziness. We excluded situations like inflammatory arthritis, simultaneous bilateral TKA, or unilateral TKA combined with implant removal, since they could potentially result in increased patient discomfort, requiring a higher dose of analgesics to achieve the same pain relief.

Further research is warranted to clarify the efficacy and adverse effects of different combinations of analgesia modalities. This will help determine their role in pain control. In future studies, it would also be beneficial to include younger and older age groups, explore different anesthesia methods, or investigate more complex TKA surgeries, to gain a comprehensive understanding of the advantages and disadvantages of MA compared to PCEA under various conditions. Additionally, future studies should compare postoperative functional scales, quality of life questionnaires, patient satisfaction questionnaires, and incidence of chronic pain. We aimed to understand inpatient effects and gain insight into the potential impact of these analgesic methods on postoperative functional recovery, the possibility of developing chronic pain, and quality of life in the future.

## 5. Conclusions

The MA strategy achieved a comparable level of postoperative pain relief as PCEA within the initial 24 h after surgery but resulted in fewer instances of motor blockade and numbness starting from 24 h postoperatively compared to PCEA. Thus, the MA strategy can be considered an innovative and safe analgesic strategy for patients undergoing primary TKA for initiating early rehabilitation while preserving effective initial pain control. On the other hand, the PCEA group exhibited better pain relief from 24 to 72 h postoperatively. Therefore, PCEA may be suitable for patients undergoing complex joint surgeries who require more comprehensive pain control without the need for early ambulation. Further prospective, randomized, long-term, and large-scale studies are necessary to determine the roles of various analgesic routes in pain control and compare the functional recovery, patient satisfaction, or quality of life between the MA and PCEA strategies.

## Figures and Tables

**Table 2:** Follow-up schedule.

Time of Visit	Definition
POD 0V1	The first visit after surgery on OP day
POD 0V2	The second visit after surgery on OP day
POD 1V1	The first visit on the first day after surgery
POD 1V2	The second visit on the first day after surgery
POD 2	The visit on the second day after surgery

The interval between the first and second visits was approximately 6 h.

**Table 3:** Demographic data between the groups.

	MA (*n* = 31)	PCEA (*n* = 87)	*p*-Value
Sex ^a^ (cases (%))			0.804
Female	25 (80.65%)	66 (75.86%)	
Male	6 (19.35%)	21 (24.14%)	
Age ^b^	69.35 ± 8.24	70.34 ± 7.05	0.523
Weight ^b^	63.63 ± 12.33	67.15 ± 10.87 (*n* = 86)	0.139
Height ^b^	153.81 ± 7.62	153.57 ± 7.07	0.874
BMI ^b^	26.90 ± 5.08	28.47 ± 5.27	0.113
Kellgren–Lawrence grade ^a^ (cases (%))			0.759
III	2 (6.45%)	9 (10.34%)	
IV	29 (93.55%)	78 (89.66%)	

^a^ Chi-square test; ^b^ Independent *t*-test.

**Table 4:** Pain intensity in the different visits between the groups.

	MA (*n* = 31)		PCEA (*n* = 87)		
Time of Visit	Cases	NRS (Mean ± SD)	Cases	NRS (Mean ± SD)	*p* Value
POD0_1 NRS-Rest	26	0.23 ± 1.17	56	0.17 ± 0.93	0.829
NRS-Motion	26	0.25 ± 1.27	63	0.09 ± 0.59	0.436
POD0_2 NRS-Rest	25	0.2 ± 1	75	0.22 ± 0.98	0.930
NRS-Motion	26	0.40 ± 1.56	66	0.31 ± 1.23	0.764
POD1_1 NRS-Rest	22	0.95 ± 1.69	51	0.54 ± 1.63	0.339
NRS-Motion	16	2.28 ± 2.66	47	1.29 ± 1.97	0.190
POD1_2 NRS-Rest	22	1.18 ± 1.83	73	0.24 ± 0.85	0.030
NRS-Motion	14	3.42 ± 2.87	42	2.01 ± 2.39	0.074
POD2 NRS-Rest	26	0.90 ± 1.69	83	0.18 ± 0.86	0.047
NRS-Motion	15	3.73 ± 2.62	55	2.02 ± 2.32	0.017

## Data Availability

All the data will be available upon motivated request to the corresponding author of the present paper.

## Abbreviations

BMI	body mass index
FNB	femoral NB
IVPCA	intravenous patient-controlled analgesia
MA	multimodal analgesic
NB	nerve block
NRS	numeric rating scale
NSAIDs	non-steroidal anti-inflammatory drugs
PAI	periarticular injection
PCEA	patient-controlled epidural analgesia
PODs	postoperative days
PONV	postoperative nausea/vomiting
ROM	range of motion
TKA	total knee arthroplasty
